# KRSA: An R package and R Shiny web application for an end-to-end upstream kinase analysis of kinome array data

**DOI:** 10.1371/journal.pone.0260440

**Published:** 2021-12-17

**Authors:** Erica A. K. DePasquale, Khaled Alganem, Eduard Bentea, Nawshaba Nawreen, Jennifer L. McGuire, Tushar Tomar, Faris Naji, Riet Hilhorst, Jaroslaw Meller, Robert E. McCullumsmith

**Affiliations:** 1 Division of Hematology, Brigham and Women’s Hospital, Boston, Massachusetts, United States of America; 2 Harvard Medical School, Boston, Massachusetts, United States of America; 3 Broad Institute of MIT and Harvard, Boston, Massachusetts, United States of America; 4 Department of Neurosciences, University of Toledo College of Medicine, Toledo, Ohio, United States of America; 5 Neuro-Aging & Viro-Immunotherapy, Center for Neurosciences, Vrije Universiteit Brussel, Brussels, Belgium; 6 Department of Neuroscience, University of Cincinnati, Cincinnati, Ohio, United States of America; 7 Department of Neurosurgery, University of Cincinnati, Cincinnati, Ohio, United States of America; 8 PamGene International B.V., s’-Hertogenbosch, The Netherlands; 9 Tercen Data Analytics Ltd, Co Waterford, Ireland; 10 Division of Biomedical Informatics, Cincinnati Children’s Hospital Medical Center, Cincinnati, Ohio, United States of America; 11 Department of Cancer Biology, University of Cincinnati College of Medicine, Cincinnati, Ohio, United States of America; 12 Department of Environmental Health, University of Cincinnati College of Medicine, Cincinnati, Ohio, United States of America; 13 Department of Electrical Engineering and Computing Systems, University of Cincinnati College of Medicine, Cincinnati, Ohio, United States of America; 14 Department of Informatics, Nicolaus Copernicus University, Torun, Poland; 15 Neurosciences institute, ProMedica, Toledo, Ohio, United States of America; Nathan S Kline Institute, UNITED STATES

## Abstract

Phosphorylation by serine-threonine and tyrosine kinases is critical for determining protein function. Array-based platforms for measuring reporter peptide signal levels allow for differential phosphorylation analysis between conditions for distinct active kinases. Peptide array technologies like the PamStation12 from PamGene allow for generating high-throughput, multi-dimensional, and complex functional proteomics data. As the adoption rate of such technologies increases, there is an imperative need for software tools that streamline the process of analyzing such data. We present Kinome Random Sampling Analyzer (KRSA), an R package and R Shiny web-application for analyzing kinome array data to help users better understand the patterns of functional proteomics in complex biological systems. KRSA is an All-In-One tool that reads, formats, fits models, analyzes, and visualizes PamStation12 kinome data. While the underlying algorithm has been experimentally validated in previous publications, we demonstrate KRSA workflow on dorsolateral prefrontal cortex (DLPFC) in male (n = 3) and female (n = 3) subjects to identify differential phosphorylation signatures and upstream kinase activity. Kinase activity differences between males and females were compared to a previously published kinome dataset (11 female and 7 male subjects) which showed similar global phosphorylation signals patterns.

## Introduction

Protein phosphorylation marks one of the most important biological mechanisms that underlies various normal cellular functions, acting in complex protein-substrate networks. Phosphorylation cascades are also perturbed in many disease states [1, 2]. As a result, kinases are one of the most studied proteins given their central role in normal and abnormal cell biological mechanisms [3–6]. Kinomics, or the study of kinases and kinase signaling, has expanded from individual activity assays, with one peptide to study one kinase, to array or chip-based technology of up to 1000 reporter peptides, called kinase arrays or kinome arrays [7–9]. The selected reporter peptides are designed to cover a broad range of signaling pathways, with large numbers allowing for a better understanding of kinase interactions and global changes that occur between two states (i.e., disease, cell type). However, analyzing the data from these peptide arrays is a complex process given that several kinases can phosphorylate the same peptide and an individual kinase can phosphorylate many peptides. For these reasons, interpretation of such data is a challenging task. As the use of these kinome arrays becomes more widespread, there is an increasing need for tools that efficiently and accurately analyze these high-throughput datasets. In particular, user-friendly analytic tools are needed for nonexpert users of kinome array platforms.

Bioinformatics tools that are specifically designed to analyze kinome array datasets are beginning to emerge. One of these analytic tools is the Kinomics Toolkit, which gives users a platform for exploration of the peptide phosphorylation data but does not provide upstream kinase predictions [10]. Another tool that was designed specifically to process kinase array data is the PamgeneAnalyzeR package, though this package is primarily focused on the pre-processing steps of kinase array datasets and not the downstream analysis [11].

However, open source tools that comprehensively analyze kinome array data are nonexistent. Current approaches of analyzing kinome array data are relying on manual statistical analyses or proprietary software such as BioNavigator by PamGene (https://pamgene.com/ps12/).

Prediction of upstream kinase activity and network-based analyses provide a biologically meaningful springboard for further kinase related research. There are existing tools that aim to predict upstream kinases based on an input of enriched genes or phosphopeptides, like KEA [12] and PTM-SEA [13]. However, none of these tools are specifically designed to take raw data from PamChip datasets and run a complete analysis pipeline starting from pre-processing to visualizing kinome networks.

A common and validated approach to predicting upstream kinase activity is to analyze the differences between 1) kinases predicted to be upstream of the peptides that are differently phosphorylated between two conditions and 2) kinases predicted to be upstream of the remaining peptides on the chip [14, 15]. In a similar statistical approach, we have previously described a method which uses random sampling to identify highly active kinases from kinome array data [16–18]. Briefly, we look at overrepresented/underrepresented kinases relative to an expected distribution using random permutation sampling of peptides. This type of analysis is valuable because it separates kinases that are truly differentially active from those who are highly active globally and don’t represent a change between states.

Here we present the Kinome Random Sampling Analyzer, or KRSA, an R package which automates many of the steps described above, including parsing kinome array raw files, peptide filtering, random sampling, different visualizations, and kinase network generation. We have also developed a web-based R Shiny application, that is built on top of the KRSA R package, that allow for users with no programming skills to analyze their data. The KRSA Shiny application can be used by biologists and data scientists alike, with no knowledge of statistical software required. KRSA makes analyzing kinome array datasets accessible and eliminates much of the human workload that the previous method required.

This method has been applied to multiple datasets and predictions have been experimentally validated in our laboratory through individual kinase activity assays and inhibitor studies [16–22]. An early version of KRSA, containing only the random sampling algorithm, identified altered phosphorylation of peptides and subsequently perturbed kinase activity in the anterior cingulate cortex (ACC) between schizophrenia and control subjects [17]. This tool was also used to analyze date from frontal cortex and hippocampus of rats subjected to lateral fluid percussion as a model of traumatic brain injury (TBI) and their sham surgery counterparts to identify differences in kinase activity in these brain regions [16]. We used the platform to explore the kinase activity in cortical neurons differentiated from induced pluripotent stem cells (iPSCs) from a schizophrenia patient with a 4-bp mutation in the DISC1 gene [18]. KRSA also was used to analyze kinome signatures of genetic perturbation of NRXN1 and FURIN1 in human induced pluripotent stem cell (hiPSC)-derived neurons [19, 20]. KRSA has also been utilized to analyze the kinome signature of mice with a genetic deletion of a specific subunit of cystine/glutamate antiporter system (xCT −/− mice) [21]. Additionally, KRSA was used to investigate the unique kinomic networks of different patient-derived pancreatic ductal adenocarcinoma (PDAC) cell lines [22]. KRSA was also used to investigate mTOR kinase signaling networks in schizophrenia [23]. More recently, KRSA identified kinases potentially involved in CO2-inhalation regulated memory liability in mice [24].

Interest within the neuroscience community in defining sex differences in the brain has increased over the past several decades. Differences in kinase activity and signaling between males and females have been implicated in sex-related variations in neuronal cell survival, outcomes after brain injury, and fear extinction, among other research areas [25–27]. To demonstrate the use of KRSA, we used postmortem brain tissue from the dorsolateral prefrontal cortex (DLPFC) to investigate kinome signature differences between female and male healthy subjects. We also paired this experiment with a previously published postmortem brain kinome array study [28] to compare against our findings.

## Design and implementation

**Fig 1:**
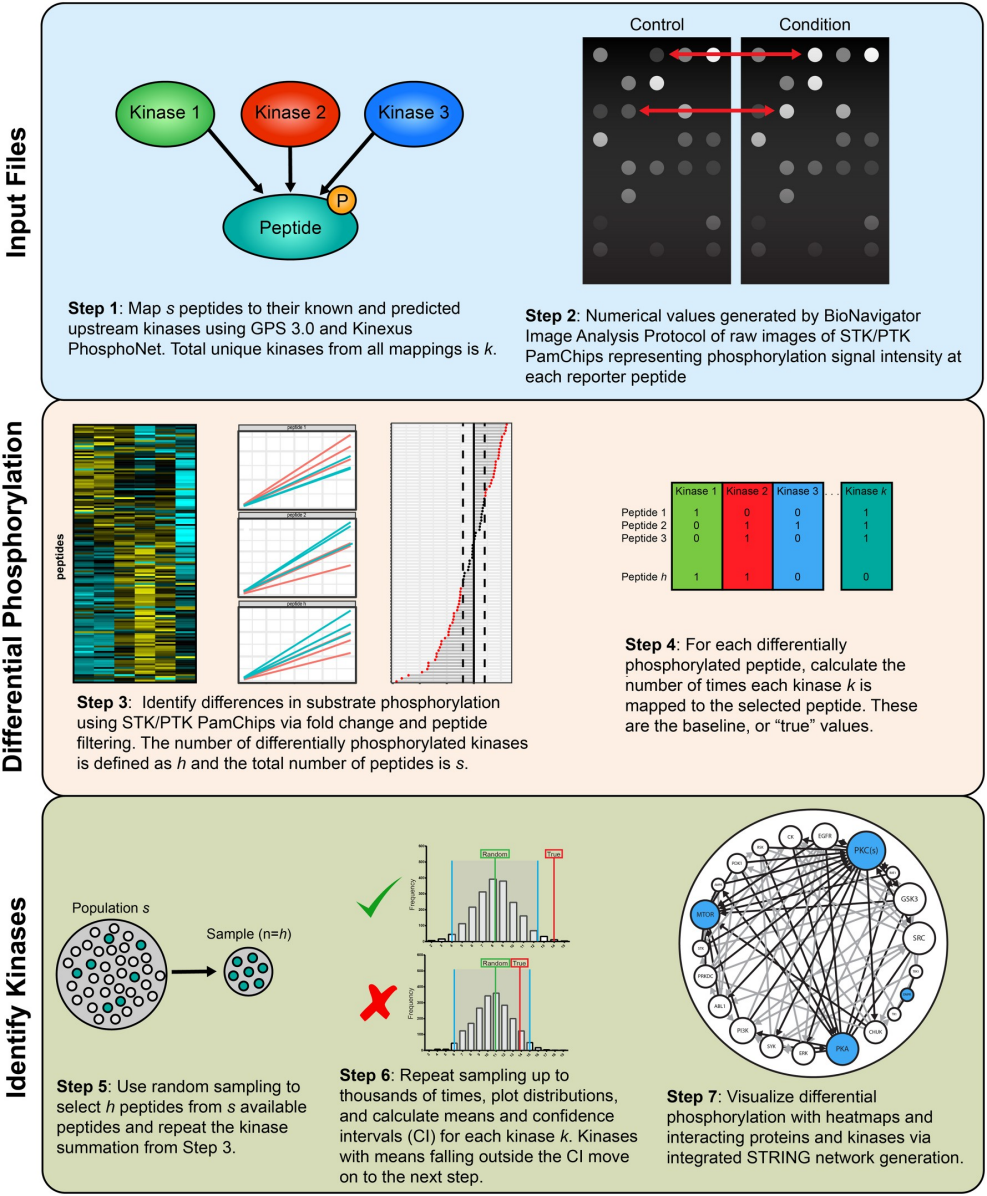
Workflow overview illustrating the primary steps of the KRSA pipeline. The “Input Files” section outlines the initial input files for KRSA, including the raw kinome array data and the peptide-kinase association file as well as the initial filtering step in KRSA. The “Identify Kinases” section describes the random sampling and distribution evaluation methods used to identify differentially active kinases. Finally, the “Expand and Validate” portion of the figure shows the kinase network generation step of KRSA and confirmation experiments that can be used to validate the predictions from KRSA.

We will briefly describe the design and functionally of the KRSA R package. More details are available in the package vignettes which are also hosted online (https://CogDisResLab.github.io/KRSA/). This resource provides comprehensive details on all KRSA functions and example datasets. The web page also hosts a complete KRSA workflow starting from reading raw data to visualizing network models. Additionally, we built an R shiny app for KRSA using the R package. The KRSA R Shiny GitHub page has complete details on how to access the app and interact with its user interface (https://github.com/CogDisResLab/KRSA_App). The general pipeline of using the KRSA R package can be divided into three main steps: loading raw files, choosing design parameters, exploring upstream kinase analysis results (Fig 1).

### Image processing and formatting

The PamChip images are pre-processed using BioNavigator to generate numerical values of the median minus background signal intensity. This raw file is then read by KRSA as the main input file. KRSA will read, parse, and reformat the raw file for downstream analyses using the *krsa_read()* function. The user then defines the groups within the samples, either using an existing variable in the input file, or creating a new one. *krsa_extractEndPointMaxExp()* and *krsa_extractEndPoint()* will extract the end point (last cycle) data points from the processed data, which then will be used to filter out some peptides based on different quality control (QC) parameters. The data will undergo a couple of QC steps using different functions: *krsa_qc_steps()* scales the negative values to the base line and optionally filters out data points with high signal saturation values. *krsa_filter_lowPeps()* will filter out peptides with very low signals. All QC steps are carried out by dedicated functions with arguments that can be adjusted by the user.

### Model fitting

Linear regression slope of the signal intensity as a function of exposure time is calculated to represent the peptide phosphorylation intensity. This value is then multiplied by 100 and log2 transformed to represent the final signal of the peptide. These steps are carried out by the *krsa_scaleModel()* function. This function will return a list of three data frames: modeled data, normalized modeled data by Barcode/Chip, and grouped modeled data. Peptides with a relatively low R^2^ can be excluded from subsequent analyses using *krsa_filter_nonLinear()*.

### Global signals visualization

The KRSA package can then plot the final signal intensity of selected samples using different figures, including heatmaps and violin plots. *krsa_heatmap()* has several data scaling options such as scaling the data by row (peptide), column (sample), and no scaling. Additionally, there is an option to specify the different algorithms that will be used for the hierarchical clustering. There is also an optional function, *krsa_cv_plot()*, that generates a coefficient of variation (CV) plot that can be used to identify potential outliers in specific groups.

### Differential phosphorylation analysis

Using the final signal values, log2 fold changes (LFCs) are calculated between different samples using two approaches. 1) across chip analysis, using the average signal across all samples and chips and 2) within chip analysis, using the log2 fold change analysis within each chip (it’s recommended to do the within chip analysis if the samples are found within each chip). We use the LFC as the main metric to determine the top differentially phosphorylated peptides using either a single cutoff or multiple cutoffs (multiple cutoffs are recommended). By doing multiple cutoff values, we address the bias in the arbitrary chosen cutoff value by doing the upstream kinase analysis on multiple peptide sets and choosing the kinase that are shown to be implicated consistently across the different peptide sets. All of that is done through *krsa_group_diff()*.

After calculating the LFCs, there are additional visualization options available. Beside the heatmaps and violin plots, users can generate a waterfall plot representing the LFCs values for each peptide using *krsa_waterfall()*. Another available figure is a curve plot, which represents the linear model fit for each peptide and colored by the different groups, which can be done by calling *krsa_curve_plot()*.

### Upstream kinase analysis

Protein kinases predicted to act on phosphorylation sites within the array peptide sequences were identified using GPS 3.0 and Kinexus Phosphonet (Kinexus Bioinformatics) [29]. These programs provide predictions for serine-threonine kinases targeting peptide sequences ordered by likelihood of binding. The union of the highest ranked 5 kinases in Kinexus and kinases with scores more than two times the prediction threshold in GPS 3.0 were considered predicted kinases for each peptide and used in KRSA analysis [18]. This list was combined with kinases shown in the literature to act on the phosphorylation sites of the peptides via PhosphoELM (http://phospho.elm.eu.org) and PhosphoSite Plus (https://www.phosphosite.org). The user has the option to use the KRSA built-in curated kinase-substrate mapping files or upload their own mapping files to perform the upstream kinase analysis. The upstream kinase analysis is done through the main function of the KRSA package, *krsa()*, which takes in the list of differentially phosphorylated peptides and kinase-substrate mapping data frame and performs the random sampling analysis. Additionally, arguments can be adjusted like the number of iterations and seed number. The *krsa()* function can be run in a parallel fashion across different cores utilizing the *future_map()* function from the furrr package (https://davisvaughan.github.io/furrr/), which will speed up the process of computation specifically when doing multiple peptide sets.

### Kinase network model

The complexity of cellular signaling ensures that kinases do not act in isolation, but instead as part of an interacting network with other kinases and proteins that regulate biological processes [30]. The nature of this system means that final KRSA predictions should include potentially interacting kinase families for downstream pathway analysis and hypothesis generation. To accomplish this goal, KRSA connects the initial set of kinase hits with other kinases using protein-protein interaction (PPI) databases. The *krsa_ball_model()* is used to generate the network, which utilized the igraph package (https://igraph.org/).

## Results

To elucidate differences in kinase activity between the brains of healthy male and female human subjects, we used KRSA to predict differential upstream kinase activity.

### Input and parameters

The input file format is a crosstab view generated by BioNavigator “Image Analysis” Protocol (PamGene International B.V.). The first line in this format comprises of meta information about the BioNavigator software version, date, and quantification type used to process the raw data. The required quantification type is either “Median_SigmBg” or “Mean_SigmBg”, which are short for “Median/or Mean Signal Minus Background”. These values represent the signal intensity of each peptide at different cycles and exposure times. Next, the following few lines contain information on the chip wells (including sample name and chip IDs) and datapoints (including cycle and exposure time). The following lines include information on the reporter peptides (including peptide ID, sequence, Uniprot Accession ID) and signal values. The main parameters found in KRSA are the minimum signal (threshold used to filter out peptides with low signals), *R*^2^ (threshold used to filter out peptides with weak linear fit), log2 fold change cutoffs (threshold used to determine differential phosphorylated peptides), and Z score cutoff (threshold used to determine kinase hits). A critical parameter is the mapping file which will be used as the reference for the upstream kinase analysis. It must contain the reporter peptides IDs and their upstream kinases (a curated mapping file is attached with the KRSA package). Input files, selected parameters, and the full script used in the analysis of this manuscript are found in the KRSA manuscript GitHub page (https://github.com/kalganem/krsa_manuscript).

### Data description

To demonstrate the various functionalities of KRSA, we set out to compare kinase activity levels between female and male dorsolateral prefrontal cortex (DLPFC). We analyzed postmortem tissue obtained from 3 male and 3 female control subjects (for demographics, see S1 and S2 Tables). We also analyzed a previously published kinome array dataset that studies the changes in protein kinase activity during Alzheimer’s Disease (AD) pathogenesis [28]. This postmortem study was performed using hippocampal (HPC) brain section samples. From this dataset, we reanalyzed all of data for the control samples (Braak Stages 0–1) for both female and male subjects. Given the Braak Stage 0 samples only contain male subjects and the apparent effect of Braak Staging on the kinome signatures, we limited ourselves to samples with Braak Stage 1, and that resulted into having 18 subjects, 11 female and 7 male (for demographics, see S1 and S2 Tables).

### Outputs

#### Global serine-threonine protein kinase activity in female vs. male (DLPFC)

**Fig 2:**
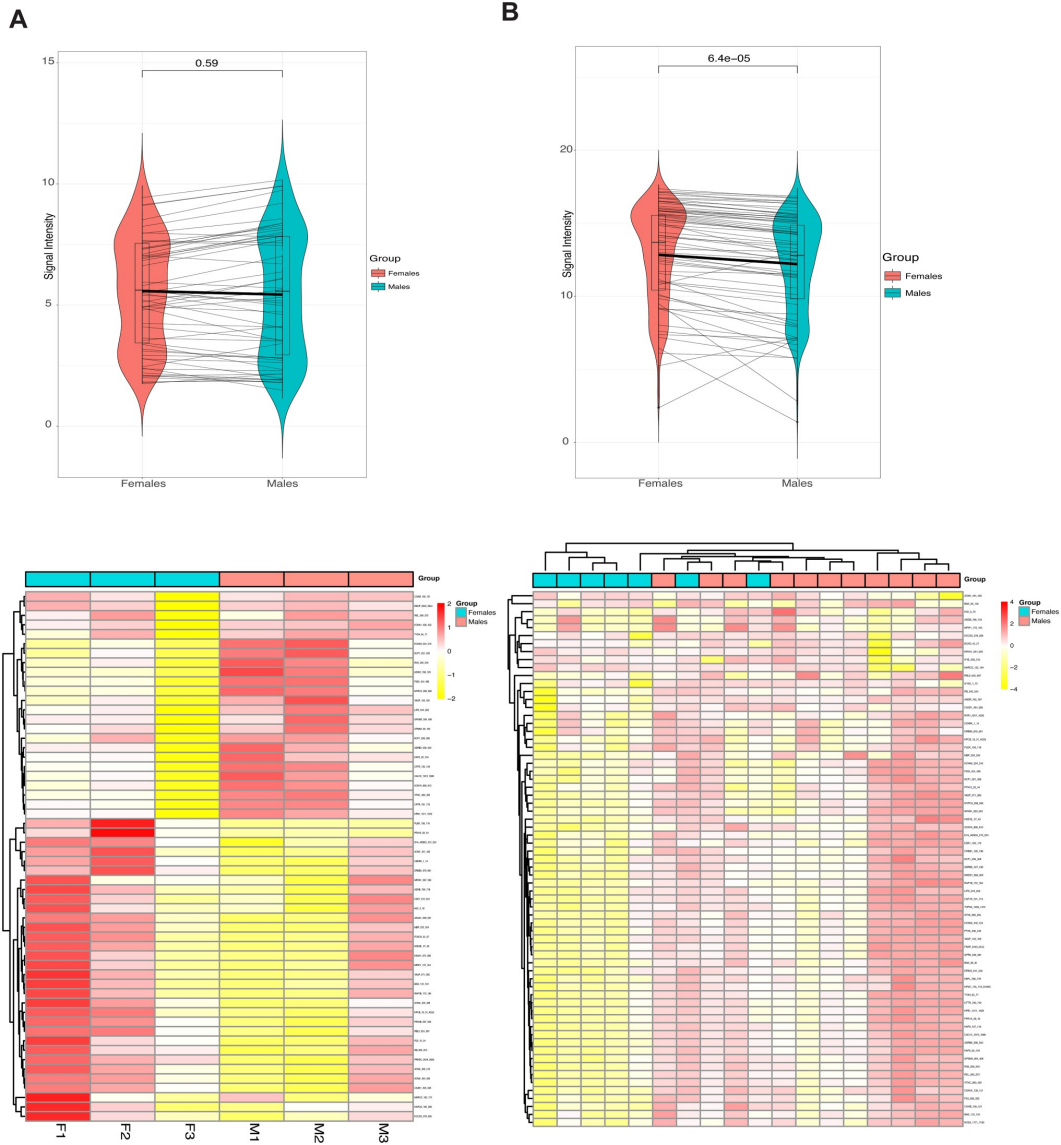
Global serine-threonine kinase activity in female vs. male DLPFC. (A) Global phosphorylation plots, showing signal intensity (phosphorylation levels) at each reporter peptide, as well as the average phosphorylation values (thick line), and a Mann-Whitney test when comparing females to males. A heatmap generated by KRSA depicting the relative signal intensity at each reporter peptide for the 6 samples on the array (3 females and 3 males) (B) Global phosphorylation plots, showing signal intensity (phosphorylation levels) at each reporter peptide, as well as the average phosphorylation values (thick line), and a Mann-Whitney test when comparing females to males using 18 control samples from the HPC cohort (11 females and 7 males). To highlight differences, the heatmap is normalized per row to present relative changes at each individual peptide between the groups. Red indicates relatively higher levels of phosphorylation and yellow indicates relatively lower levels of phosphorylation.

In the QC steps, KRSA filtered out 55 peptides that were considered undetectable, 2 peptides that were not linearly increasing with exposure time (based on R^2^ > 0.9), and 11 references/control peptides. The log2 fold change was calculated for the remaining 84 peptides by taking the female group as the “baseline.” Based on the three chosen LFC cutoff values (0.2, 0.3, 0.4), three peptide sets were extracted with the lengths of 56, 44, and 33 respectively. Using the first set of peptides, *krsa_violin_plot_grouped()* was used to visualize the global phosphorylation levels and the results indicate that the signatures are not significantly different between females and males (Fig 2A, p value = 0.59 using a Mann-Whitney test). A heatmap of phosphorylation intensity at each reporter peptide was generated using *krsa_heatmap()* and scaled by row (by peptide) (Fig 2A).

#### Global serine-threonine protein kinase activity of the independent dataset (HPC)

The same approach was done using the independent cohort. However, since this cohort has a larger number of subjects, we were able to detect a significant difference between male and female kinome signatures (Fig 2B, p value = 6.04e-5 using a Mann-Whitney test). The samples signatures showed distinct clustering between males and females in both the heatmap unsupervised clustering (Fig 2B) and in the principal component analysis (PCA) (S1 Fig).

#### Altered kinase activity in female vs. male (DLPFC)

**Fig 3:**
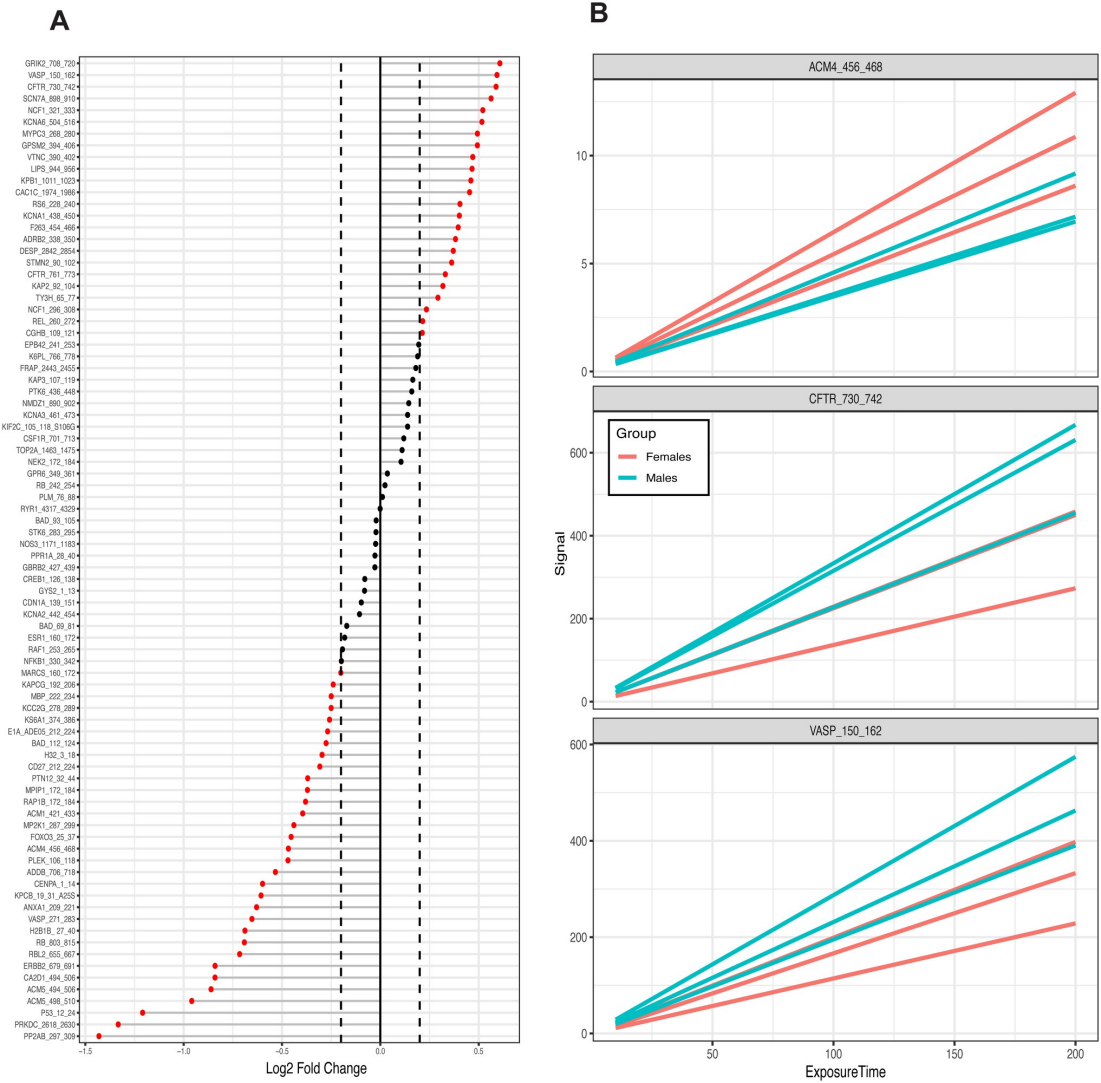
Changes in phosphorylation at reporter peptides in female vs. male DLPFC. (A) Waterfall plot showing log2 fold changes (LFC) in phosphorylation at reporter peptides for female vs. male DLPFC. Peptides with positive LFC indicate higher phosphorylation in males, and peptides with negative LFC indicates lower phosphorylation in males. The dashed line indicates the 0.2 LFC cutoff (B) Representative examples of linear model fit of the phosphorylation curves of three reporter peptides in female vs. male DLPFC.

**Fig 4:**
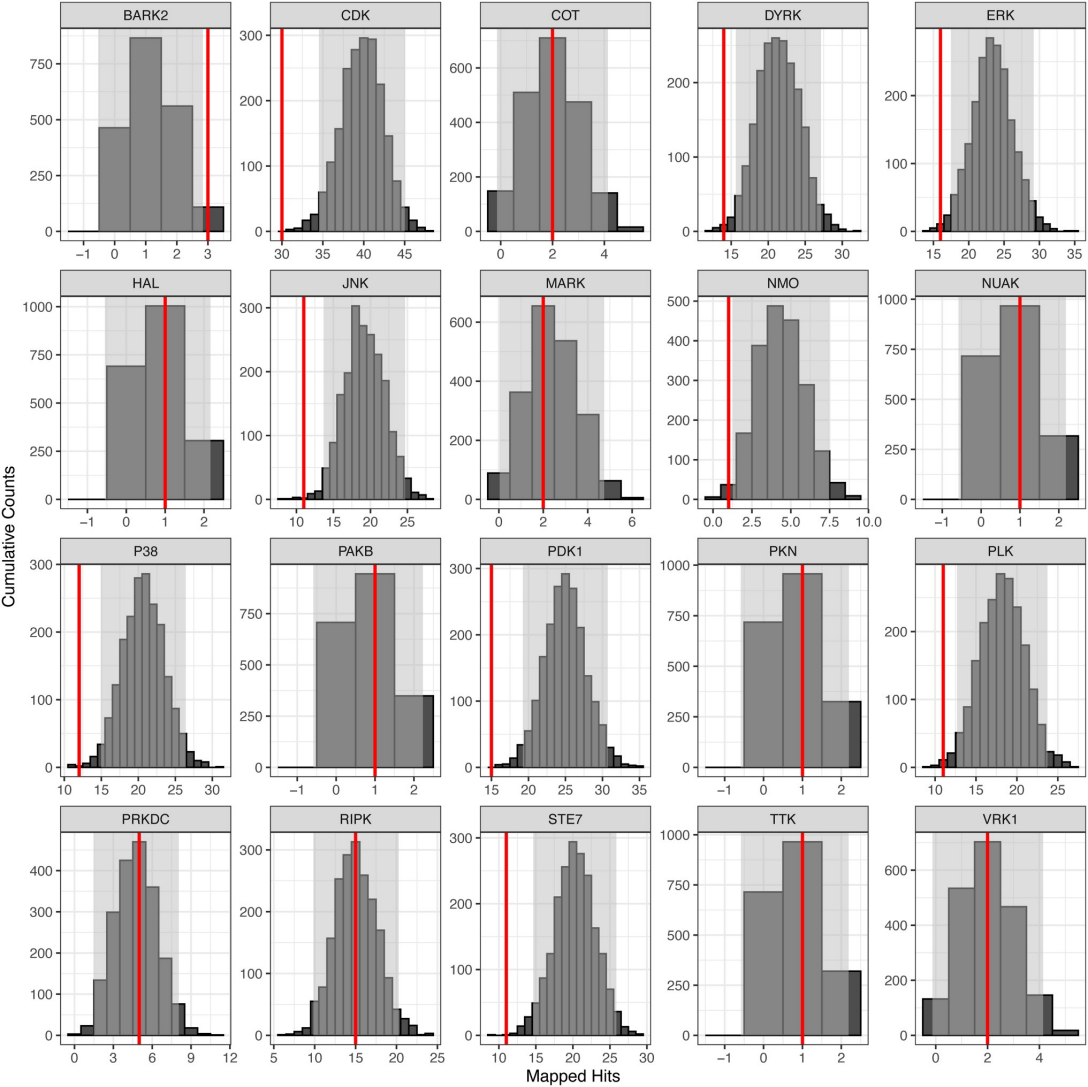
Observed frequency of selected kinases relative to expected random sampling distribution in female vs. male DLPFC. Examples are shown for kinases identified in the reporter peptides more than by random chance alone (e.g., BARK2), less than by random chance (e.g., ERK, CDK, JNK), as well as for kinases that were not identified to be significantly relevant (e.g., COT, MARK, RIPK). KRSA was performed with 2000 iterations and histograms were automatically generated. Gray areas indicate ± 2 standard deviations from the expected distribution mean. The red line indicates the number of mapped peptides for the corresponding kinase based on one of the log2 fold change cutoffs (0.2 shown here). The Z scores for each kinase are derived by calculating how many standard deviations the red line is away from the mean.

The different peptide sets, based on the different LFC cutoff values, were used to perform the upstream kinase analysis step in KRSA. *krsa_waterfall()* was used to visualize the average LFCs at each peptide (Fig 3A). A small set of peptides were chosen to demonstrate the output of *krsa_curve_plot()*, which shows the linear fit models (Fig 3B). *krsa()* was used to run the upstream kinases analysis which generates data frames that contain random sampling distribution, standard deviation, and Z scores for each kinase family for each peptide set. The Z scores were averaged to determine the final score of each kinase family (S2 Fig). Taking one peptide set as an example, the top kinases include CDK, PDK1, STE7, and in addition to others included in Table 1. Additionally, *krsa_histogram()* was used to visualize the experimental peptide hits relative to the random sampling distribution (Fig 4).

**Table 1:** Predicted kinases and distributions for female vs. male (DLPFC).

Kinase	Observed	SamplingAvg	SD	Z
**CDK**	30	39.76	2.62	-3.72
**PDK1**	15	24.97	2.86	-3.48
**STE7**	11	20.26	2.80	-3.30
**P38**	12	20.69	2.85	-3.05
**JNK**	11	19.19	2.75	-2.98
**PLK**	11	18.21	2.76	-2.61
**DYRK**	14	21.38	2.88	-2.56
**ERK**	16	23.36	2.91	-2.53
**BARK2**	3	1.16	0.84	2.19
**NMO**	1	4.38	1.55	-2.18

#### Altered kinase activity in female vs. male in the independent dataset (HPC)

We used a similar method to determine the upstream kinase hits for the HPC cohort, and one set of peptides led to the identification of serval different serine-threonine kinase families differentially represented in HPC between female and male control subjects (Table 2).

**Table 2:** Predicted kinases and distributions for female vs. male (HPC).

Kinase	Observed	SamplingAvg	SD	Z
**PKA**	42	28.55	2.97	4.52
**JNK**	10	22.79	2.90	-4.41
**ERK**	15	28.05	3.00	-4.35
**P38**	13	24.59	2.89	-4.01
**RSK**	51	39.43	2.98	3.88
**DMPK**	59	48.99	2.59	3.86
**DYRK**	15	25.76	2.86	-3.77
**PDK1**	19	29.90	2.92	-3.73
**PKG**	33	22.89	2.84	3.56
**AKT**	28	18.99	2.74	3.29

To statistically compare the similarities between the two cohorts, DLPFC (current study) and HPC (Rosenberger et al.), we performed a Pearson correlation analysis of upstream kinase hits (using the KRSA Z scores). The results of this analysis showed a significant correlation between these two studies (r = 0.65, *P* = 5.9e-11) (S5 Fig). For the HPC cohort, and due to the larger sample size, the unsupervised clustering and PCA also showed a clear separation between male and female signatures. Using Z-score threshold of 2, we saw an overlap of several kinase families, including STE7, JNK, ERK, DYRK, P38, and PDK1 among the two cohorts. There were kinases that were unique in each cohort like CDK and PEK in the DLPFC dataset, and PKA and AKT in the HPC dataset.

#### Kinase network model of female vs. male DLPFC

**Fig 5:**
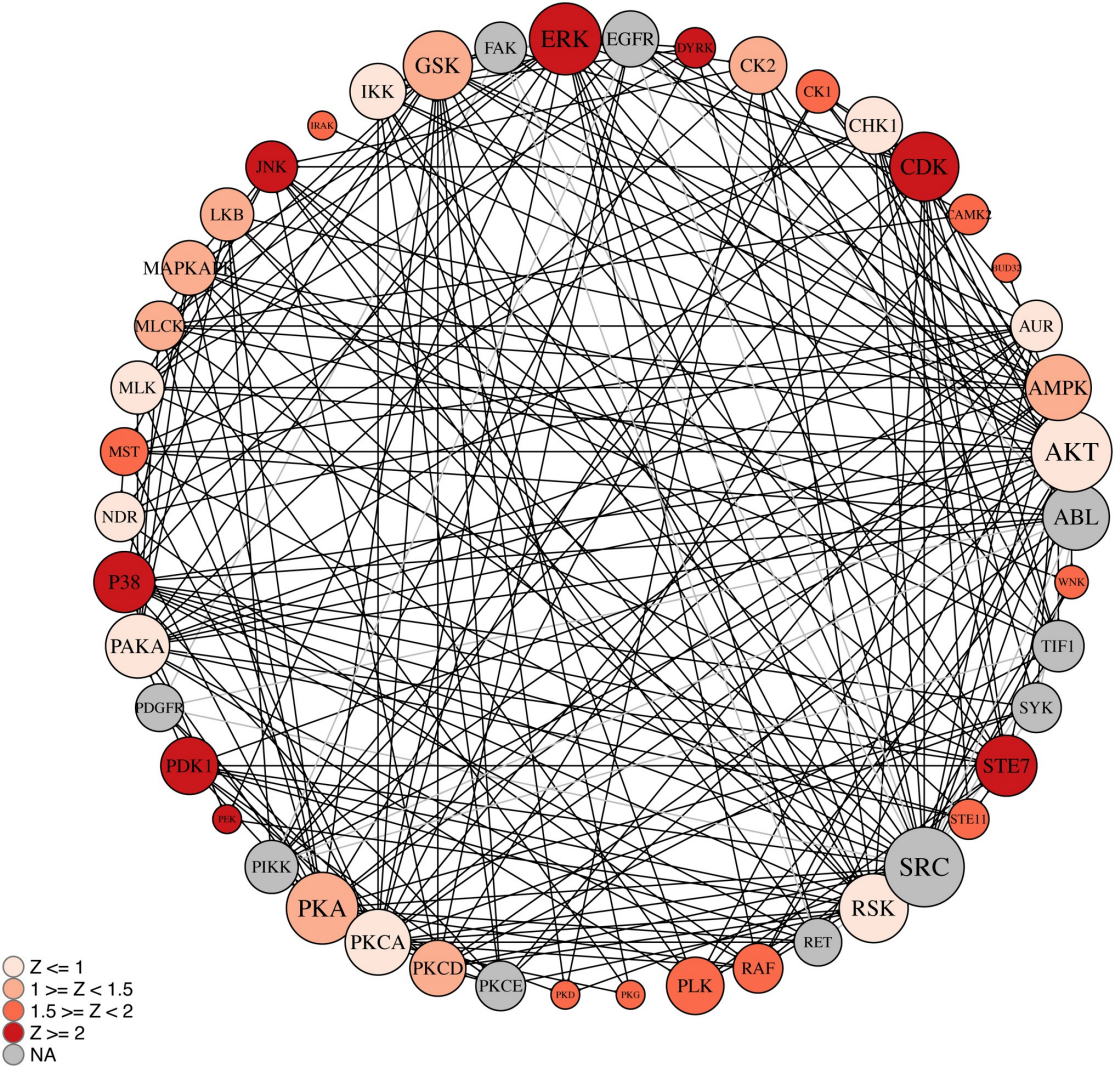
Kinase network model of female vs. male DLPFC. The kinase network was obtained in KRSA by growing the kinome array hits with kinase interacting partners as identified using STRING and PhosphoSitePlus. The kinome array hits are color coded by the averaged z score values. Circle size corresponds to the number of interactions, with larger circles having more interactions. Black lines represent interactions with a kinome array direct hit, while gray represent interactions made between associated the other kinase families.

The *krsa_ball_model()* function was used to generate a network to connect the kinase hits with other kinases families (Fig 5). One of the largest nodes in this pathway based on number of connections is ERK, which is also one of the highest scoring hits identified by KRSA. ERK has an observed presence in our data much lower than would be expected by random chance (16 versus 23.36, Z-score = -2.53). The model also highlights potential targets that are important in the network despite their low Z-scores. For example, even though the Z-score for AKT is low (Z-score = -0.719), it connects to many of the nodes in the kinase network model suggesting its important role within the network.

#### Validation of robustness

To test the robustness of detecting similar upstream kinase scores under different signal-to-noise ratios (SNRs), we added random noise (unseeded) to the ratio of differences between the tested groups in our existing data based on different SNR values (from 1 to 30). We then ran our standard upstream kinase analysis under each of these conditions. We calculated the Pearson correlation coefficient of the kinase Z scores generated by KRSA between our original analysis and the generated noisy data. The correlation coefficient ranged from 0.74 and 0.98, showing stronger correlation (above 0.9) when the SNR is larger than 4 (S4 Fig).

## Discussion

Unlike many diseases and conditions, where distinct high-magnitude changes in gene expression and subsequent downstream function occur because of the disease processes, differences between healthy male and female brains are theoretically subtler and harder to characterize. As an example of KRSA’s capabilities, we probed for kinase activity differences in the male and female brain using postmortem dorsolateral prefrontal cortex. We also compared our findings to a previously published kinome array study [28]. The samples from that study showed a similar pattern of changes between female and male kinome signatures as an overall higher phosphorylation levels in the female samples (Fig 2B, *P* < 0.05). Examining the global signal, the sum average of the phosphorylation signal intensity of all reporter peptides, showed differences between males and females.

Comparing the results from the upstream kinases analysis and network modeling, there are common kinase hits between the two cohorts such as MAPKs (ERK, JNK, P38) and AKTs. ERKs, or extracellular signal-regulated kinases, are part of the MAPK/ERK pathway that regulates a wide variety of processes including differentiation, proliferation, adhesion, and migration, among others [31]. ERKs have sex-related differential activity, as kinases of this family are involved in regulating hormones [32, 33]. The activation of estrogen receptor beta (ERβ) induces the MAPK pathway which explains the differences between males and females that we observed in the KRSA results [34]. AKT, also known a protein kinase B, is in part activated by hormonal regulation and takes part in processes such as metabolism, apoptosis, and proliferation [35]. Recent research has identified sex-specific differences in AKT isoforms as a key factor in regulating neurobiological processes [36]. AKT was also differentially phosphorylated in hippocampal samples in the same direction as KRSA predictions [37]. Finally, another study identified differential expression of AKT-associated genes, but did not find significance in phosphorylation of AKT itself between males and females [38]. Instead, similar levels of AKT phosphorylation were maintained in the brain between the sexes through differing level of PTEN expression [38]. This last example highlights the power of the kinase network model versus examining kinases alone, as KRSA can draw attention to highly connected nodes that are near or below the threshold for being considered a hit but are still potentially important targets to study due to their importance within the network.

In the area of kinomics, there is a need for end-to-end processing of kinome array data in a user-friendly, open source, and interactive environment. The Kinome Random Sampling Analyzer (KRSA) R package and Shiny app fill this gap in the field and serve as a steppingstone for the use and interpretation of kinome array data for laboratory biologists and computational biologists alike.

## Supporting information

S1 Fig Principal Component Analysis (PCA) of the independent dataset from the HPC cohort. Using the subjects in HPC cohort dataset (controls only) showing the clustering of samples and the factors that most explain the variance in the kinome signatures. PMI: postmortem interval, Barcode: Chip ID.
(TIF)

S2 Fig Z scores waterfall plot for the DLPFC cohort. Multiple z scores for each kinase that were calculated using the different peptide sets (that were derived based on the different log2 fold change cutoffs, 0.2, 0.3, and 0.4). The bigger dots represent the averaged Z scores which are also color coded based on the absolute values of the averaged z scores.
(TIF)

S3 Fig Venn diagrams showing overlap of the total of overrepresented/underrepresented kinases for both cohorts. DLPFC from current study, and the HPC cohort study. Filtered kinase with absolute values of Z scores equal or above 2 for both datasets. DLPFC: dorsolateral prefrontal cortex, HPC: hippocampus.
(TIF)

S4 Fig Correlation analysis between the DLPFC and HPC cohorts. To statistically compare the similarities between the two cohorts, DLPFC (current study) and HPC (Rosenberger et al.), a Pearson correlation analysis of upstream kinase hits (using the KRSA Z scores) is performed. DLPFC: dorsolateral prefrontal cortex, HPC: hippocampus.
(TIF)

S5 Fig Robustness analysis of capturing upstream kinase scores under different signal-to-noise ratio (SNR) values. To detect the upstream kinase scores under different SNRs, we added random noise (unseeded) to the ratios of differences between the tested groups in our existing data based on different SNR values (from 1 to 30), and ran our proposed upstream kinase analysis under each condition. We calculated the Pearson correlation coefficient of the kinase Z scores generated by KRSA between our original analysis and the noisy data.
(TIF)

S1 Table Kinome array subject demographics of the DLPFC cohort. Subjects demographics of the DLPFC cohort (current study) indicating sex, age, pH, and PMI. pH: acidity measure, PMI: postmortem interval.
(DOCX)

S2 Table Kinome array subject demographics for the HPC cohort. Subjects demographics of the HPC cohort (Rosenberger et al.) indicating sex, age, and PMI (control subjects only). pH: acidity measure, PMI: postmortem interval.
(DOCX)

## References

[1] Simpson CM, Zhang B, Hornbeck PV, Gnad F. Systematic analysis of the intersection of disease mutations with protein modifications. BMC Med Genomics. 2019;12(Suppl 6):109. doi: 10.1186/s12920-019-0543-2 31345222 PMC6657027

[2] Hanahan D, Weinberg RA. Hallmarks of cancer: the next generation. Cell. 2011;144(5):646–74. doi: 10.1016/j.cell.2011.02.013 21376230

[3] Ardito F, Giuliani M, Perrone D, Troiano G, Lo Muzio L. The crucial role of protein phosphorylation in cell signaling and its use as targeted therapy (Review). Int J Mol Med. 2017;40(2):271–80. doi: 10.3892/ijmm.2017.3036 28656226 PMC5500920

[4] Ubersax JA, Ferrell JE Jr.. Mechanisms of specificity in protein phosphorylation. Nat Rev Mol Cell Biol. 2007;8(7):530–41. doi: 10.1038/nrm2203 17585314

[5] Pawson T, Scott JD. Protein phosphorylation in signaling—50 years and counting. Trends Biochem Sci. 2005;30(6):286–90. doi: 10.1016/j.tibs.2005.04.013 15950870

[6] Lahiry P, Torkamani A, Schork NJ, Hegele RA. Kinase mutations in human disease: interpreting genotype-phenotype relationships. Nat Rev Genet. 2010;11(1):60–74. doi: 10.1038/nrg2707 20019687

[7] Baharani A, Trost B, Kusalik A, Napper S. Technological advances for interrogating the human kinome. Biochem Soc Trans. 2017;45(1):65–77. doi: 10.1042/BST20160163 28202660

[8] Houseman BT, Mrksich M. Towards quantitative assays with peptide chips: a surface engineering approach. Trends Biotechnol. 2002;20(7):279–81. doi: 10.1016/s0167-7799(02)01984-4 12062966

[9] Diks SH, Kok K, O’Toole T, Hommes DW, van Dijken P, Joore J, et al. Kinome profiling for studying lipopolysaccharide signal transduction in human peripheral blood mononuclear cells. The Journal of biological chemistry. 2004;279(47):49206–13. doi: 10.1074/jbc.M405028200 15355981

[10] Dussaq AM, Kennell T Jr, Eustace NJ, Anderson JC, Almeida JS, Willey CD. Kinomics toolbox-A web platform for analysis and viewing of kinomic peptide array data. PLoS One. 2018;13(8):e0202139. doi: 10.1371/journal.pone.0202139 30130366 PMC6103510

[11] Bekkar A, Nasrallah A, Guex N, Fajas L, Xenarios I, Lopez-Mejia IC. PamgeneAnalyzeR: open and reproducible pipeline for kinase profiling. Bioinformatics. 2020. doi: 10.1093/bioinformatics/btz858 31922550 PMC7755406

[12] Lachmann A, Ma’ayan A. KEA: kinase enrichment analysis. Bioinformatics. 2009;25(5):684–6. doi: 10.1093/bioinformatics/btp026 19176546 PMC2647829

[13] Krug K, Mertins P, Zhang B, Hornbeck P, Raju R, Ahmad R, et al. A Curated Resource for Phosphosite-specific Signature Analysis. Mol Cell Proteomics. 2019;18(3):576–93. doi: 10.1074/mcp.TIR118.000943 30563849 PMC6398202

[14] Anderson JC, Duarte CW, Welaya K, Rohrbach TD, Bredel M, Yang ES, et al. Kinomic exploration of temozolomide and radiation resistance in Glioblastoma multiforme xenolines. Radiother Oncol. 2014;111(3):468–74. doi: 10.1016/j.radonc.2014.04.010 24813092 PMC4119546

[15] Isayeva T, Xu J, Ragin C, Dai Q, Cooper T, Carroll W, et al. The protective effect of p16(INK4a) in oral cavity carcinomas: p16(Ink4A) dampens tumor invasion-integrated analysis of expression and kinomics pathways. Mod Pathol. 2015;28(5):631–53. doi: 10.1038/modpathol.2014.149 25523612

[16] Dorsett CR, McGuire JL, Niedzielko TL, DePasquale EA, Meller J, Floyd CL, et al. Traumatic Brain Injury Induces Alterations in Cortical Glutamate Uptake without a Reduction in Glutamate Transporter-1 Protein Expression. J Neurotrauma. 2017;34(1):220–34. doi: 10.1089/neu.2015.4372 27312729 PMC5198172

[17] McGuire JL, Depasquale EA, Funk AJ, O’Donnovan SM, Hasselfeld K, Marwaha S, et al. Abnormalities of signal transduction networks in chronic schizophrenia. NPJ Schizophr. 2017;3(1):30. doi: 10.1038/s41537-017-0032-6 28900113 PMC5595970

[18] Bentea E, Depasquale EAK, O’Donovan SM, Sullivan CR, Simmons M, Meador-Woodruff JH, et al. Kinase network dysregulation in a human induced pluripotent stem cell model of DISC1 schizophrenia. Mol Omics. 2019;15(3):173–88. doi: 10.1039/c8mo00173a 31106784 PMC6563817

[19] Flaherty E, Zhu S, Barretto N, Cheng E, Deans PJM, Fernando MB, et al. Neuronal impact of patient-specific aberrant NRXN1alpha splicing. Nat Genet. 2019;51(12):1679–90. doi: 10.1038/s41588-019-0539-z 31784728 PMC7451045

[20] Schrode N, Ho SM, Yamamuro K, Dobbyn A, Huckins L, Matos MR, et al. Synergistic effects of common schizophrenia risk variants. Nat Genet. 2019;51(10):1475–85. doi: 10.1038/s41588-019-0497-5 31548722 PMC6778520

[21] Bentea E, Villers A, Moore C, Funk AJ, O’Donovan SM, Verbruggen L, et al. Corticostriatal dysfunction and social interaction deficits in mice lacking the cystine/glutamate antiporter. Mol Psychiatry. 2020. doi: 10.1038/s41380-020-0751-3 32366950 PMC7609546

[22] Creeden JF, Alganem K, Imami AS, Brunicardi FC, Liu SH, Shukla R, et al. Kinome Array Profiling of Patient-Derived Pancreatic Ductal Adenocarcinoma Identifies Differentially Active Protein Tyrosine Kinases. Int J Mol Sci. 2020;21(22). doi: 10.3390/ijms21228679 33213062 PMC7698519

[23] Chadha R, Alganem K, McCullumsmith RE, Meador-Woodruff JH. mTOR kinase activity disrupts a phosphorylation signaling network in schizophrenia brain. Mol Psychiatry. 2021. doi: 10.1038/s41380-021-01135-9 33990769

[24] Lin B, Alganem K, O’Donovan SM, Jin Z, Naghavi F, Miller OA, et al. Activation of acid-sensing ion channels by carbon dioxide regulates amygdala synaptic protein degradation in memory reconsolidation. Mol Brain. 2021;14(1):78. doi: 10.1186/s13041-021-00786-7 33962650 PMC8106190

[25] Armstead WM, Riley J, Vavilala MS. Sex and Age Differences in Epinephrine Mechanisms and Outcomes after Brain Injury. J Neurotrauma. 2017;34(8):1666–75. doi: 10.1089/neu.2016.4770 27912253 PMC5397223

[26] Matsuda S, Matsuzawa D, Ishii D, Tomizawa H, Sutoh C, Shimizu E. Sex differences in fear extinction and involvements of extracellular signal-regulated kinase (ERK). Neurobiol Learn Mem. 2015;123:117–24. doi: 10.1016/j.nlm.2015.05.009 26079214

[27] Zhang L, Li PP, Feng X, Barker JL, Smith SV, Rubinow DR. Sex-related differences in neuronal cell survival and signaling in rats. Neurosci Lett. 2003;337(2):65–8. doi: 10.1016/s0304-3940(02)01179-5 12527389

[28] Rosenberger AF, Hilhorst R, Coart E, Garcia Barrado L, Naji F, Rozemuller AJ, et al. Protein Kinase Activity Decreases with Higher Braak Stages of Alzheimer’s Disease Pathology. J Alzheimers Dis. 2016;49(4):927–43. doi: 10.3233/JAD-150429 26519433 PMC4927853

[29] Xue Y, Liu Z, Gao X, Jin C, Wen L, Yao X, et al. GPS-SNO: computational prediction of protein S-nitrosylation sites with a modified GPS algorithm. PloS one. 2010;5(6):e11290. doi: 10.1371/journal.pone.0011290 20585580 PMC2892008

[30] Yao Z, Petschnigg J, Ketteler R, Stagljar I. Application guide for omics approaches to cell signaling. Nat Chem Biol. 2015;11(6):387–97. doi: 10.1038/nchembio.1809 25978996

[31] Busca R, Pouyssegur J, Lenormand P. ERK1 and ERK2 Map Kinases: Specific Roles or Functional Redundancy? Front Cell Dev Biol. 2016;4:53. doi: 10.3389/fcell.2016.00053 27376062 PMC4897767

[32] Brown JL, Xie J, Brieno-Enriquez MA, Sones JL, Angulo CN, Boehm U, et al. Sex- and Age-Specific Impact of ERK Loss Within the Pituitary Gonadotrope in Mice. Endocrinology. 2018;159(3):1264–76. doi: 10.1210/en.2017-00653 29300908 PMC5802804

[33] Pergola C, Dodt G, Rossi A, Neunhoeffer E, Lawrenz B, Northoff H, et al. ERK-mediated regulation of leukotriene biosynthesis by androgens: a molecular basis for gender differences in inflammation and asthma. Proceedings of the National Academy of Sciences of the United States of America. 2008;105(50):19881–6. doi: 10.1073/pnas.0809120105 19064924 PMC2597692

[34] Mizuno K, Giese KP. Towards a molecular understanding of sex differences in memory formation. Trends in neurosciences. 2010;33(6):285–91. doi: 10.1016/j.tins.2010.03.001 20356635

[35] Nicholson KM, Anderson NG. The protein kinase B/Akt signalling pathway in human malignancy. Cellular signalling. 2002;14(5):381–95. doi: 10.1016/s0898-6568(01)00271-6 11882383

[36] Wong H, Levenga J, LaPlante L, Keller B, Cooper-Sansone A, Borski C, et al. Isoform-specific roles for AKT in affective behavior, spatial memory, and extinction related to psychiatric disorders. Elife. 2020;9. 10.7554/eLife.56630 PMC7787664 33325370

[37] Sheppard PAS, Puri TA, Galea LAM. Sex differences and estradiol effects in MAPK and Akt cell signalling across subregions of the hippocampus. Neuroendocrinology. 2021. doi: 10.1159/000519072 34407537

[38] de Mello NP, Andreotti DZ, Orellana AM, Scavone C, Kawamoto EM. Inverse sex-based expression profiles of PTEN and Klotho in mice. Sci Rep. 2020;10(1):20189. doi: 10.1038/s41598-020-77217-5 33214645 PMC7677532

