# Supplementary figures and images for: KRSA: An R package and R Shiny web application for an end-to-end upstream kinase analysis of kinome array data
Source: PLoS One. 2021 Dec 17;16(12):e0260440. doi: 10.1371/journal.pone.0260440 (PMC8682895; doi:10.1371/journal.pone.0260440)

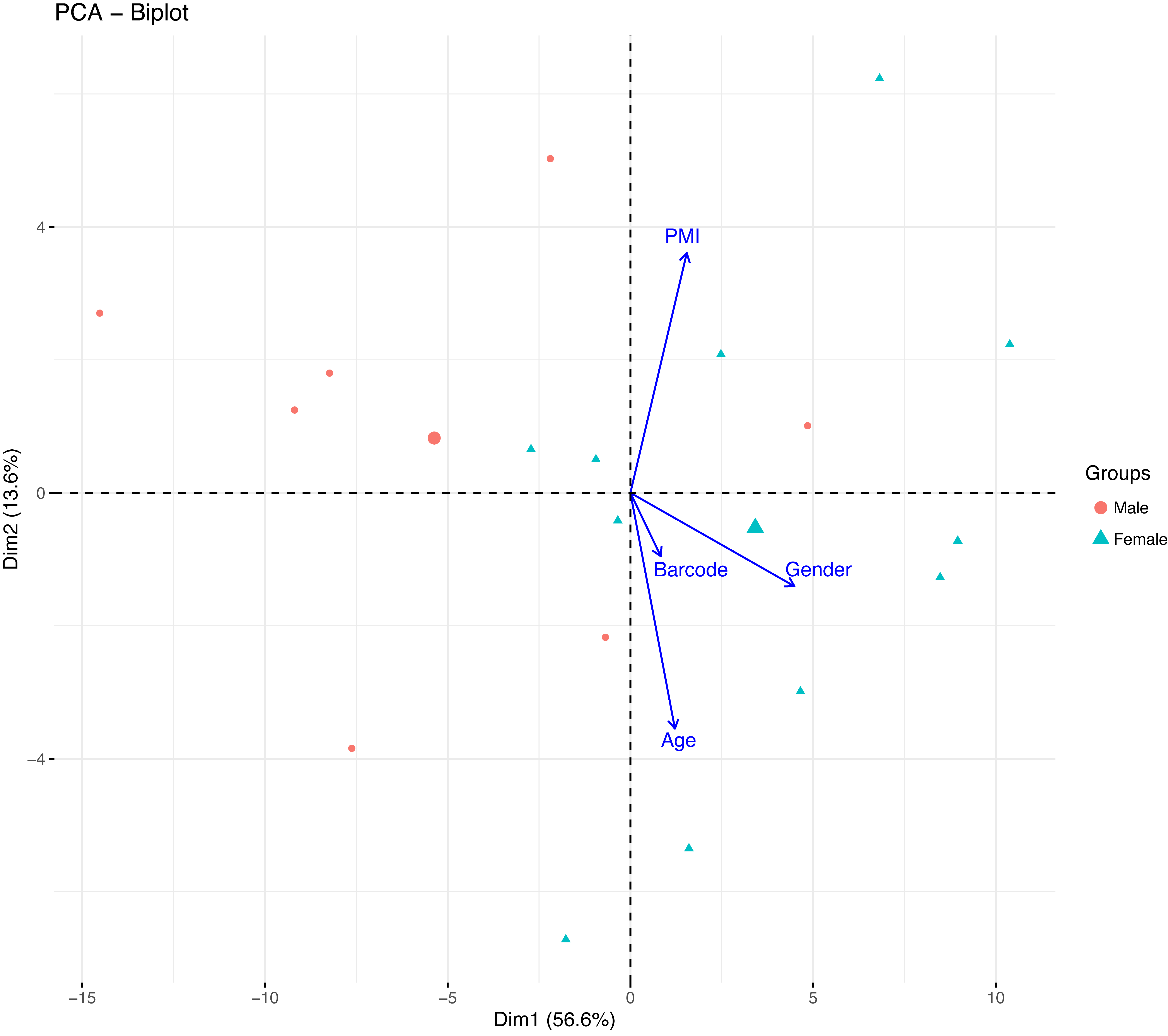

Supplement: S1 Fig — Using the subjects in HPC cohort dataset (controls only) showing the clustering of samples and the factors that most explain the variance in the kinome signatures. PMI: postmortem interval, Barcode: Chip ID. (TIF) [file pone.0260440.s001.tif]

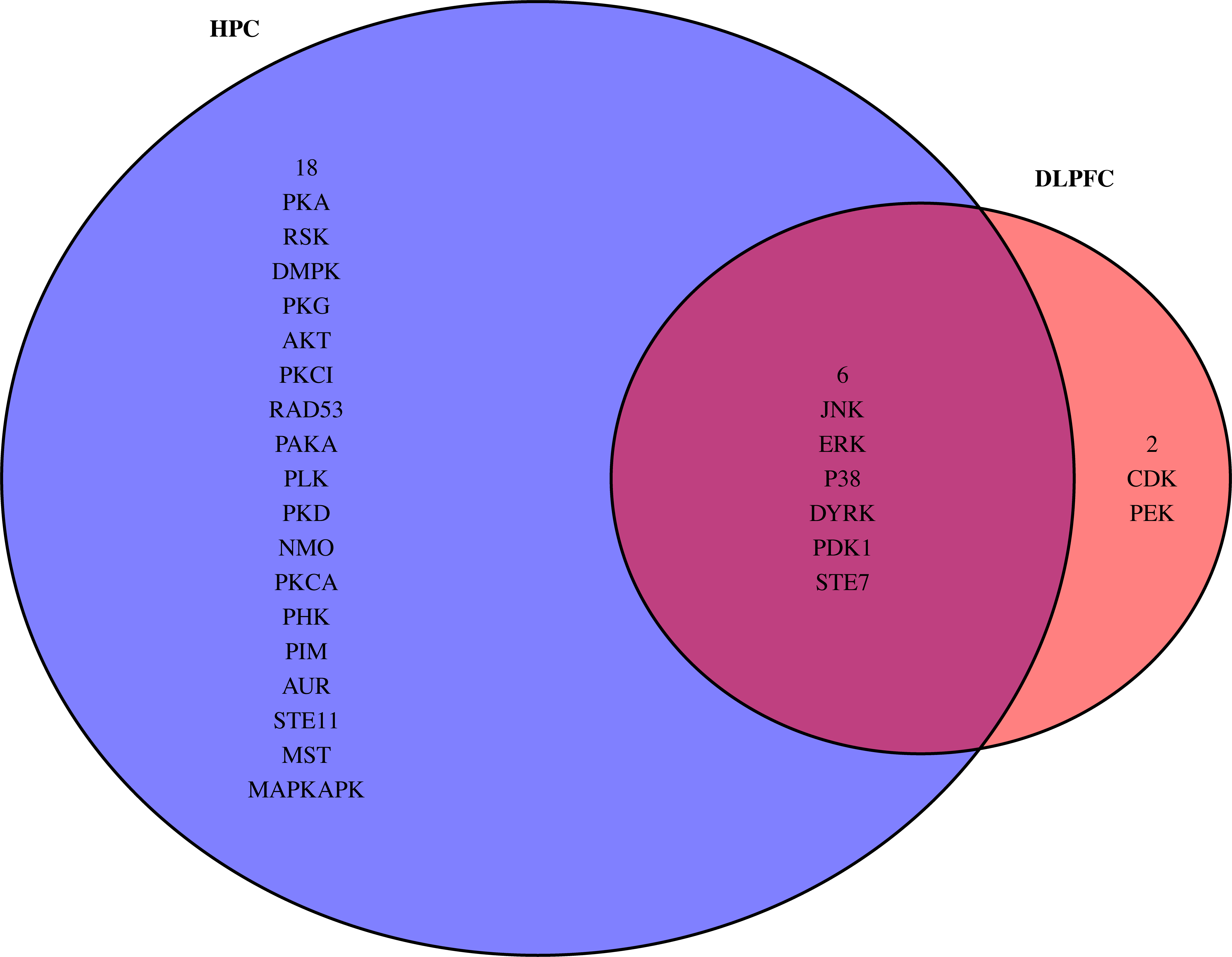

Supplement: S3 Fig — DLPFC from current study, and the HPC cohort study. Filtered kinase with absolute values of Z scores equal or above 2 for both datasets. DLPFC: dorsolateral prefrontal cortex, HPC: hippocampus. (TIF) [file pone.0260440.s003.tif]

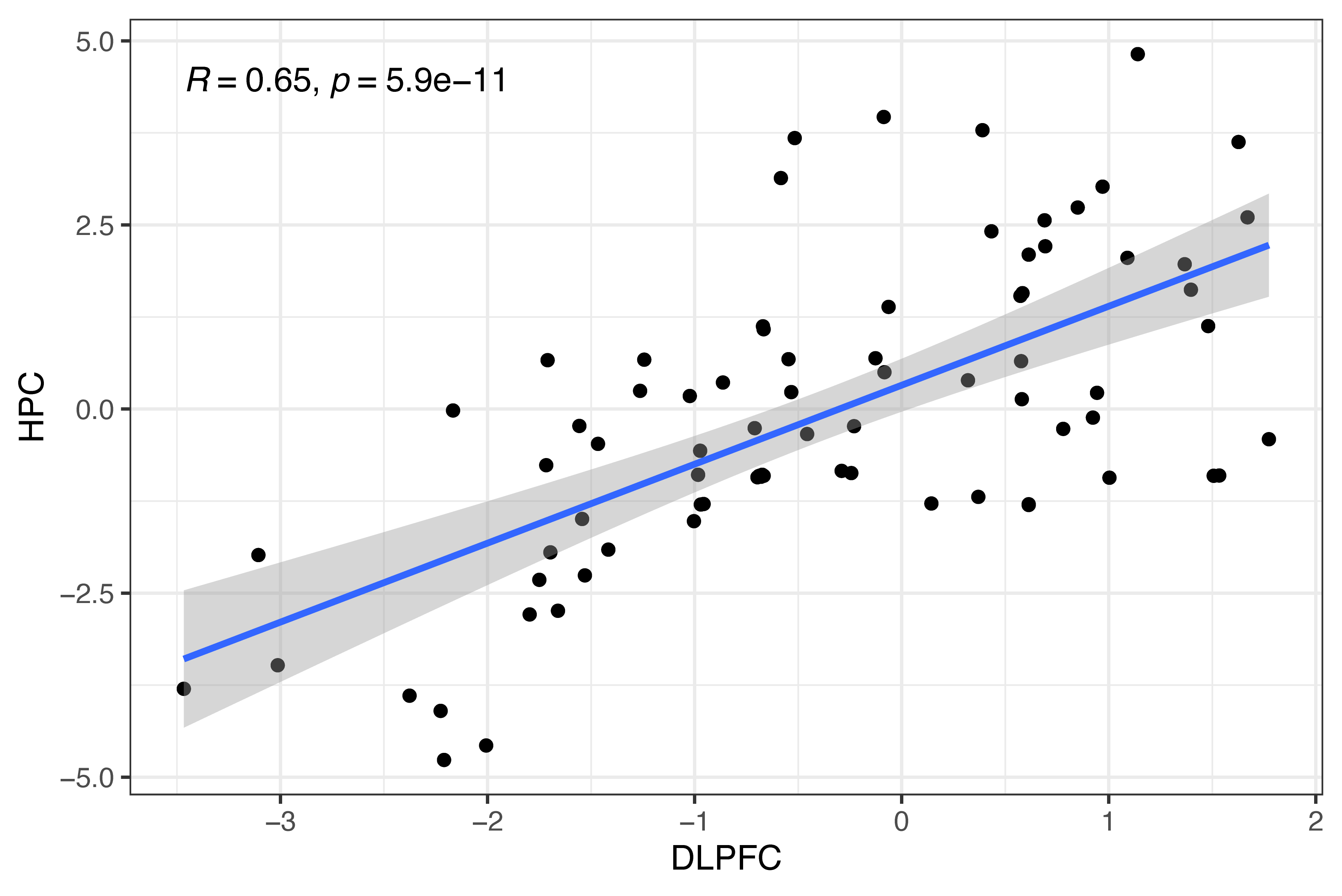

Supplement: S4 Fig — To statistically compare the similarities between the two cohorts, DLPFC (current study) and HPC (Rosenberger et al.), a Pearson correlation analysis of upstream kinase hits (using the KRSA Z scores) is performed. DLPFC: dorsolateral prefrontal cortex, HPC: hippocampus. (TIF) [file pone.0260440.s004.tif]

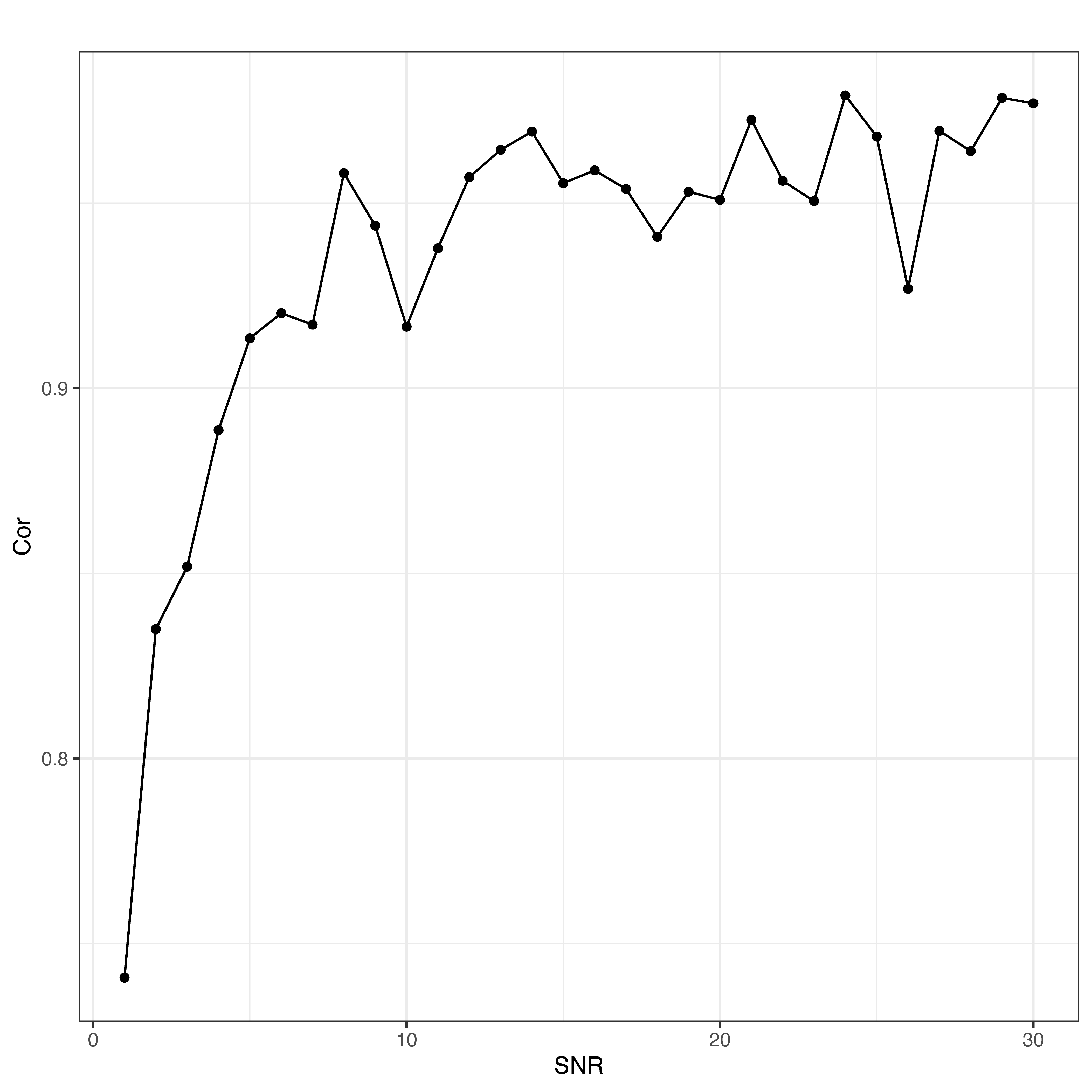

Supplement: S5 Fig — To detect the upstream kinase scores under different SNRs, we added random noise (unseeded) to the ratios of differences between the tested groups in our existing data based on different SNR values (from 1 to 30), and ran our proposed upstream kinase analysis under each condition. We calculated the Pearson correlation coefficient of the kinase Z scores generated by KRSA between our original analysis and the noisy data. (TIF) [file pone.0260440.s005.tif]
