# Supplementary material for: KRSA: An R package and R Shiny web application for an end-to-end upstream kinase analysis of kinome array data
Source: PLoS One. 2021 Dec 17;16(12):e0260440. doi: 10.1371/journal.pone.0260440 (PMC8682895; doi:10.1371/journal.pone.0260440)
Supplement: S1 Table — Subjects demographics of the DLPFC cohort (current study) indicating sex, age, pH, and PMI. pH: acidity measure, PMI: postmortem interval. (DOCX) [file pone.0260440.s006.docx]

**S1 Table.** **Kinome array subject demographics of the DLPFC cohort.** Subjects demographics of the DLPFC cohort (current study) indicating sex, age, pH, and PMI. pH: acidity measure, PMI: postmortem interval.

| Subject | Sex | Age | pH | PMI (h) |
| --- | --- | --- | --- | --- |
| M1 | M | 73 | 6.4 | 17 |
| M2 | M | 71 | 6.4 | 13 |
| M3 | M | 71 | 6.4 | 20 |
| F1 | F | 76 | 6.3 | 23 |
| F2 | F | 73 | 5.9 | 25 |
| F3 | F | 77 | 6.6 | 30 |
