# Supplementary material for: KRSA: An R package and R Shiny web application for an end-to-end upstream kinase analysis of kinome array data
Source: PLoS One. 2021 Dec 17;16(12):e0260440. doi: 10.1371/journal.pone.0260440 (PMC8682895; doi:10.1371/journal.pone.0260440)
Supplement: S2 Table — Subjects demographics of the HPC cohort (Rosenberger et al.) indicating sex, age, and PMI (control subjects only). pH: acidity measure, PMI: postmortem interval. (DOCX) [file pone.0260440.s007.docx]

**S2 Table. Kinome array subject demographics for the HPC cohort.** Subjects demographics of the HPC cohort (Rosenberger et al.) indicating sex, age, and PMI (control subjects only). pH: acidity measure, PMI: postmortem interval.

| Subject | Sex | Age | PMI (h) |
| --- | --- | --- | --- |
| TIS163 | M | 62 | 7 |
| TIS164 | M | 82 | 5.5 |
| TIS173 | M | 91 | 4 |
| TIS191 | M | 77 | 7 |
| TIS194 | M | 58 | 5 |
| TIS214 | M | 79 | 6 |
| TIS216 | M | 82 | 5 |
| TIS188 | F | 93 | 4 |
| TIS198 | F | 85 | 7 |
| TIS204 | F | 86 | 6 |
| TIS209 | F | 82 | 4 |
| TIS212 | F | 60 | 6.5 |
| TIS215 | F | 83 | 5 |
| TIS217 | F | 77 | 2.5 |
| TIS221 | F | 92 | 7 |
| TIS223 | F | 60 | 7 |
| TIS227 | F | 50 | 4 |
| TIS228 | F | 83 | 4 |
